# Supplementary material for: Evidence for two dimensional anisotropic Luttinger liquids at millikelvin temperatures
Source: Nat Commun. 2023 Nov 2;14:7025. doi: 10.1038/s41467-023-42821-2 (PMC10622557; doi:10.1038/s41467-023-42821-2)
Supplement: Supplementary file 1 — Supplementary Information [file 41467_2023_42821_MOESM1_ESM.pdf]

Supplementary Information for "Evidence for Two Dimensional Anisotropic Luttinger Liquids at Millikelvin Temperatures"

Guo Yu[1,2,#], Pengjie Wang[1,#], Ayelet J. Uzan[1], Yanyu Jia[1], Michael Onyszczak[1], Ratnadwip Singha[3], Xin Gui[3], Tiancheng Song[1], Yue Tang[1], Kenji Watanabe[4], Takashi Taniguchi[5], Robert J. Cava[3], Leslie M. Schoop[3], Sanfeng Wu[1,*]

[1]Department of Physics, Princeton University, Princeton, New Jersey 08544, USA
[2]Department of Electrical and Computer Engineering, Princeton University, Princeton, New Jersey 08544, USA
[3]Department of Chemistry, Princeton University, Princeton, New Jersey 08544, USA
[4]Research Center for Functional Materials, National Institute for Materials Science, 1-1 Namiki, Tsukuba 305-0044, Japan
[5]International Center for Materials Nanoarchitectonics, National Institute for Materials Science, 1-1 Namiki, Tsukuba 305-0044, Japan

[#] These authors contributed equally to this work
[*] Email: sanfengw@princeton.edu

This Supplementary Information file includes the following contents:

Supplementary Figure 1: Cartoon illustration of device fabrication process

Supplementary Figure 2: Comparison between transport behaviors of monolayer WTe_2, ~3° $tWTe_2$ and ~5° $tWTe_2$

Supplementary Figure 3: Behaviors of a 5° $tWTe_2$ device approaching the millikelvin regime

Supplementary Figure 4: Effect of twist angle on $tWTe_2$ devices

Supplementary Figure 5: Transport anisotropy and identifying the hard direction

Supplementary Figure 6: Transport anisotropy data for Device 2

Supplementary Figure 7: Differential resistance dV/dI measurement for a 5° $tWTe_2$ device

Supplementary Figure 8: Additional plots of conductance data

Supplementary Figure 9: Scaled conductance under different contact geometries

Supplementary Figure 10: Displacement field (D) effect on the LL behavior (Device 2)

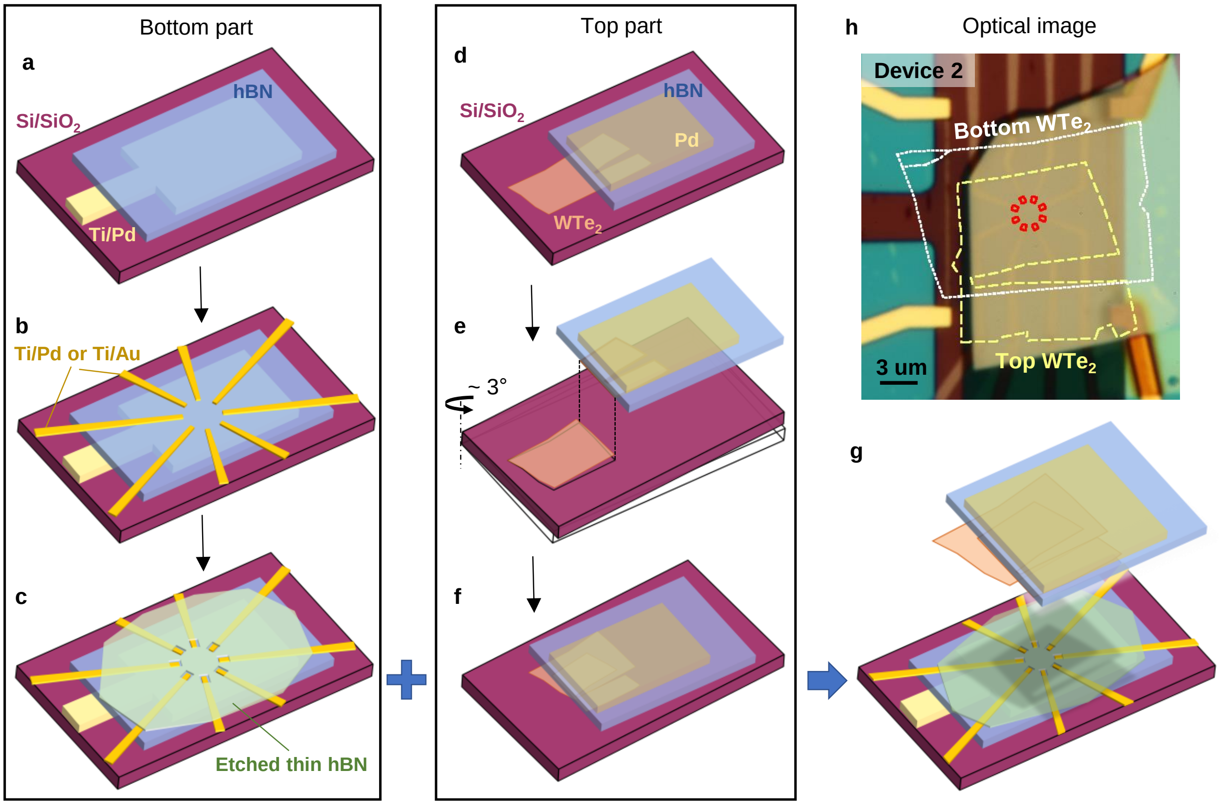

Supplementary Fig. 1 | Cartoon illustration of device fabrication process. a, Ti/Pd layer deposited on an insulating Si/SiO2 substrate, followed by transferring a layer of hBN dielectric for the bottom gate. **b,** Ti/Pd (device 1) or Ti/Au (device 2) electrodes deposited on the bottom hBN. **c,** Thin layer of hBN transferred on electrodes and selectively etched to expose only the ends of electrodes. **d,** Top hBN dielectric and pre-deposited Ti/Pd (device 1, Pd for device 2) layer were picked up and aligned with monolayer WTe_2. **e & f,** Tear-and-stack process for creating the $tWTe_2$ stack. **g**, Complete the device by stacking the top part onto the prepared bottom part. Steps **d-g** were carried out in an Ar-filled glovebox. **h,** An optical image of device 2. The yellow, white and red lines mark the top WTe_2, bottom WTe_2 and the electric contact regions, respectively.

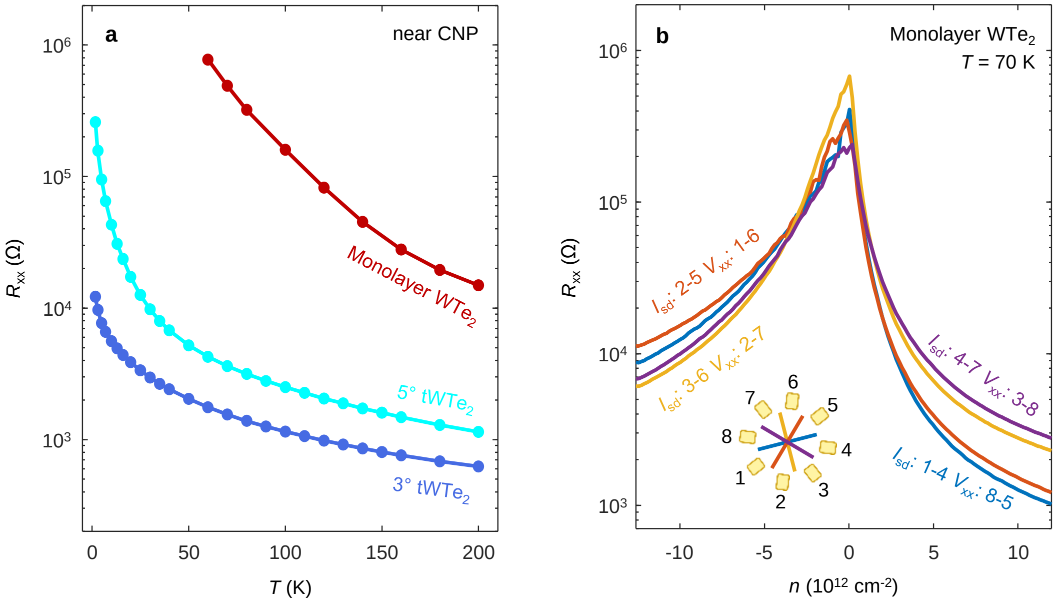

Supplementary Fig. 2| Comparison between transport behaviors of monolayer WTe_2, ~3° $tWTe_2$ and ~5° $tWTe_2$. a, Temperature dependent four-probe resistance of the three devices at a doping density near charge neutrality point (CNP). **b**, Typical Four-probe resistances of a monolayer WTe_2 device measured along different in plane directions (contact configurations are shown as inset).

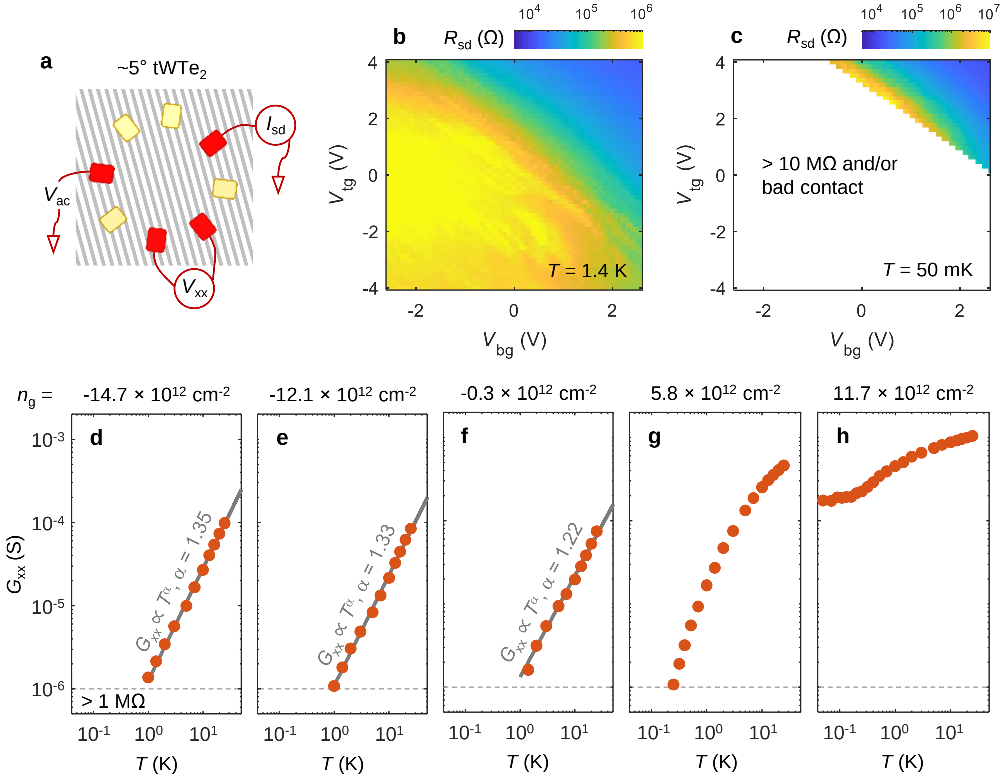

Supplementary Fig. 3| Behaviors of a 5° $tWTe_2$ device approaching the millikelvin regime. a, An illustration of the measurement configuration. **b,** Two-probe resistance $R_{sd} \equiv V_{ac}/I_{sd}$ as a function of the top and bottom gate voltages at 1.4 K. **c,** The same measurement data but at 50 mK. The white color denotes regions where resistance is too large to be reliably determined or the contact is bad. Note that only a small portion of the gate map on the electron-doping side could be measured reliably down to 50 mK. **d-h,** Temperature dependent four probe resistance $G_{xx} \equiv I_{sd}/V_{xx}$ at selected doping levels. On the hole side, the resistance quickly goes above ~ MΩ, preventing a reliable quantitative analysis of their behaviors at millikelvin.

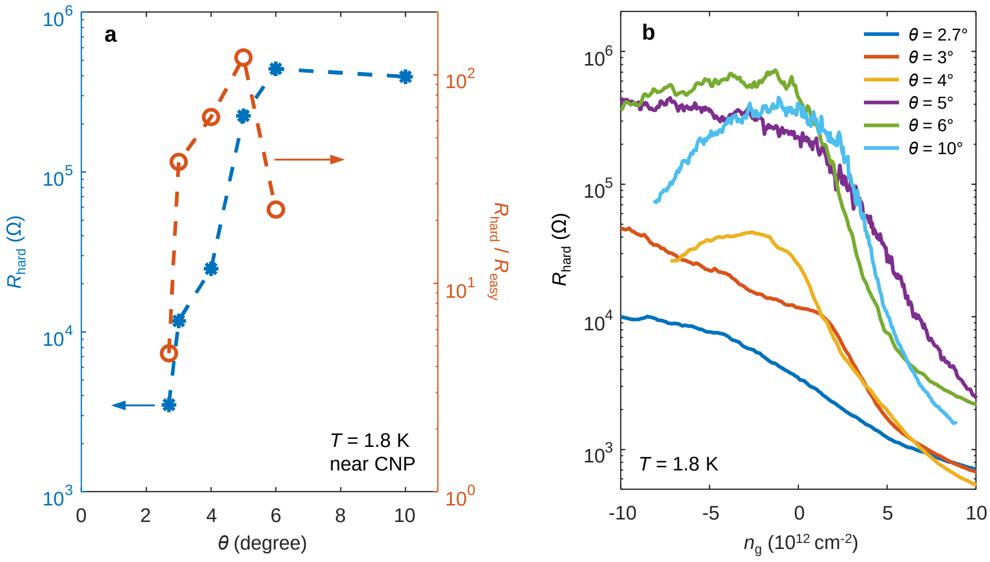

Supplementary Fig. 4| Effect of twist angle on tWTe$_2$ devices. a, measured four-probe resistance R_{hard} (blue) and extracted anisotropy R_{hard}/R_{easy} (orange) at different small twist angles ranging from 2.7^o to 10°, measured at 1.8 K near charge neutrality. For each device, the values are determined by measuring the resistance along different in-plane directions and R_{hard} is chosen as the resistance along the relatively hard direction. Note that anisotropy data for the 10° device is missing since it can't be determined due to an imperfection in this specific device. **b**, R_{hard} *versus* n_g at 1.8 K for each device.

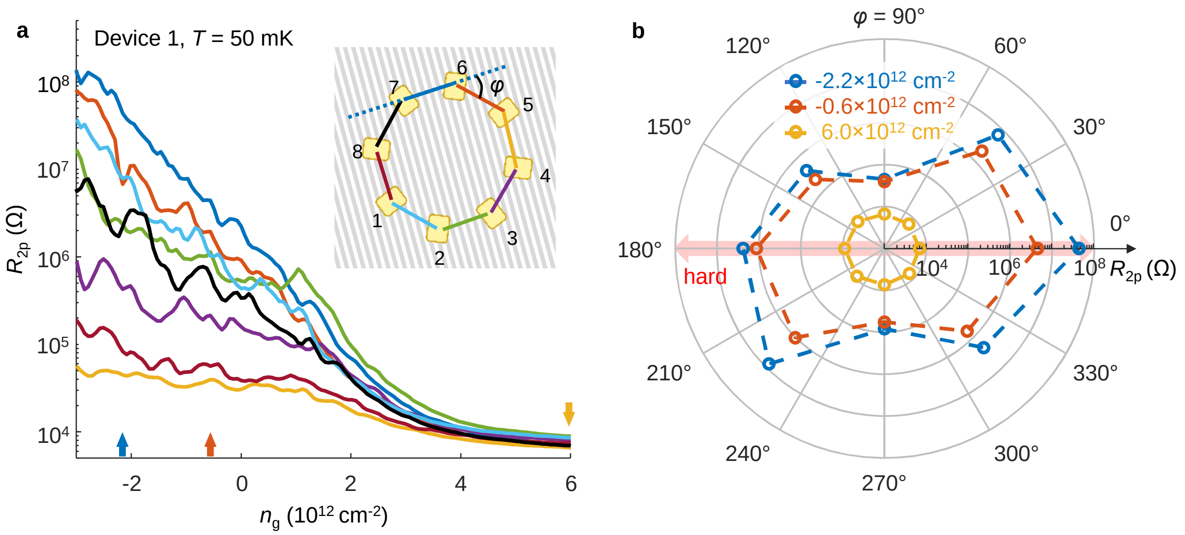

Supplementary Fig. 5 | Transport anisotropy and identifying the hard direction. a, Two-probe resistance (R_{2p}) measured between neighboring contacts as a function of n_g. Data were taken in device 1 at 50 mK. Inset illustrates the corresponding pair of probes (connected by a colored solid line) used for each curve, one-to-one matched by their colors. **b**, Identifying the hard direction by the angle dependent two-probe resistance. The direction along the contact pair of 6 and 7 is defined as the reference (0°). Resistance values for all 8 pairs (extracted from **a**) are plotted at three typical n_g, line colors correspond to the arrows in **a**. On the electrode side (yellow), no anisotropy is seen. On the hole side or near charge neutrality (blue and red), exceptionally large anisotropy is seen, and the hard direction can be clearly identified as near 0° (i.e., along contact 6 & 7). The radical axis (R_{2p}) is in log scale.

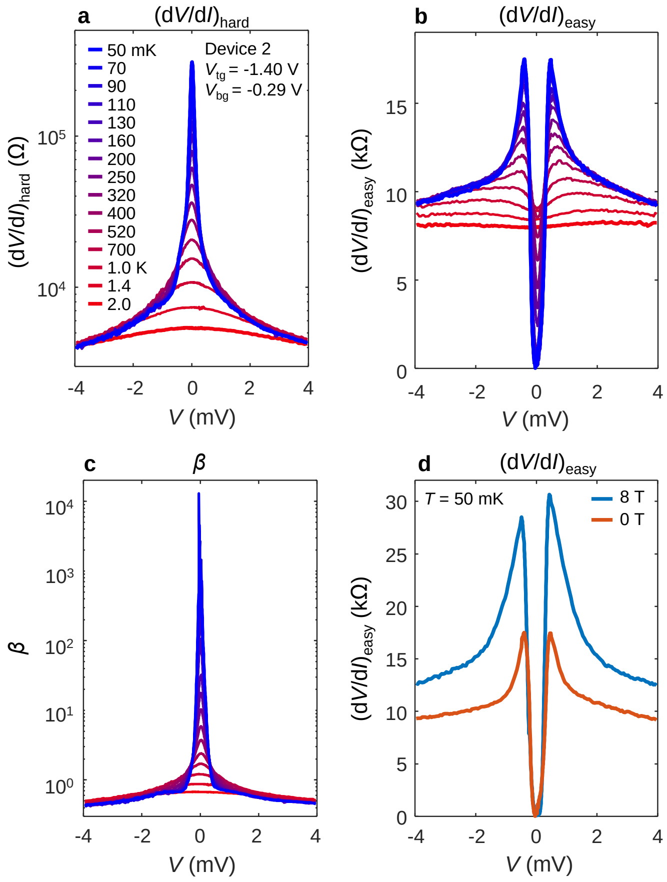

Supplementary Fig. 6 | Transport anisotropy data for device 2. a, Differential resistance dV/dI *v.s.* d.c. bias V at various T ranging from 50 mK to 2 K, measured along the hard direction. The gate configuration is chosen as V_{tg}= -1.40 V, V_{bg} = -0.29 V. **b,** The same data but for the measurement taken along the easy direction. **c,** Bias dependent anisotropy at corresponding T. **d**, Magnetic field effect on the differential resistance along the easy direction, at 50 mK.

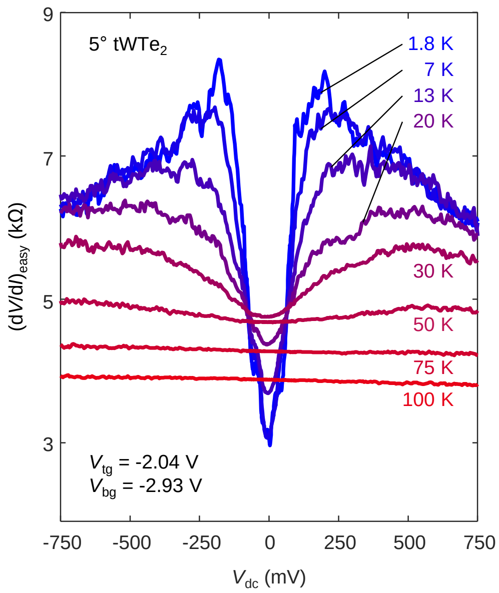

Supplementary Fig. 7| Differential resistance d*V*/d*I* measurement for a 5° $tWTe_2$ device. dV/dI measured along the easy direction at different temperatures from 1.8 K up to 100 K. A zero-bias dip is developed, similar to the ~ 3° $tWTe_2$ device reported in the main text. The difference here is that (1) the associated energy scale is larger (as seen by the wider dip in the bias axis) and (2) the zero-bias value doesn't reach zero in this case at 1.8 K.

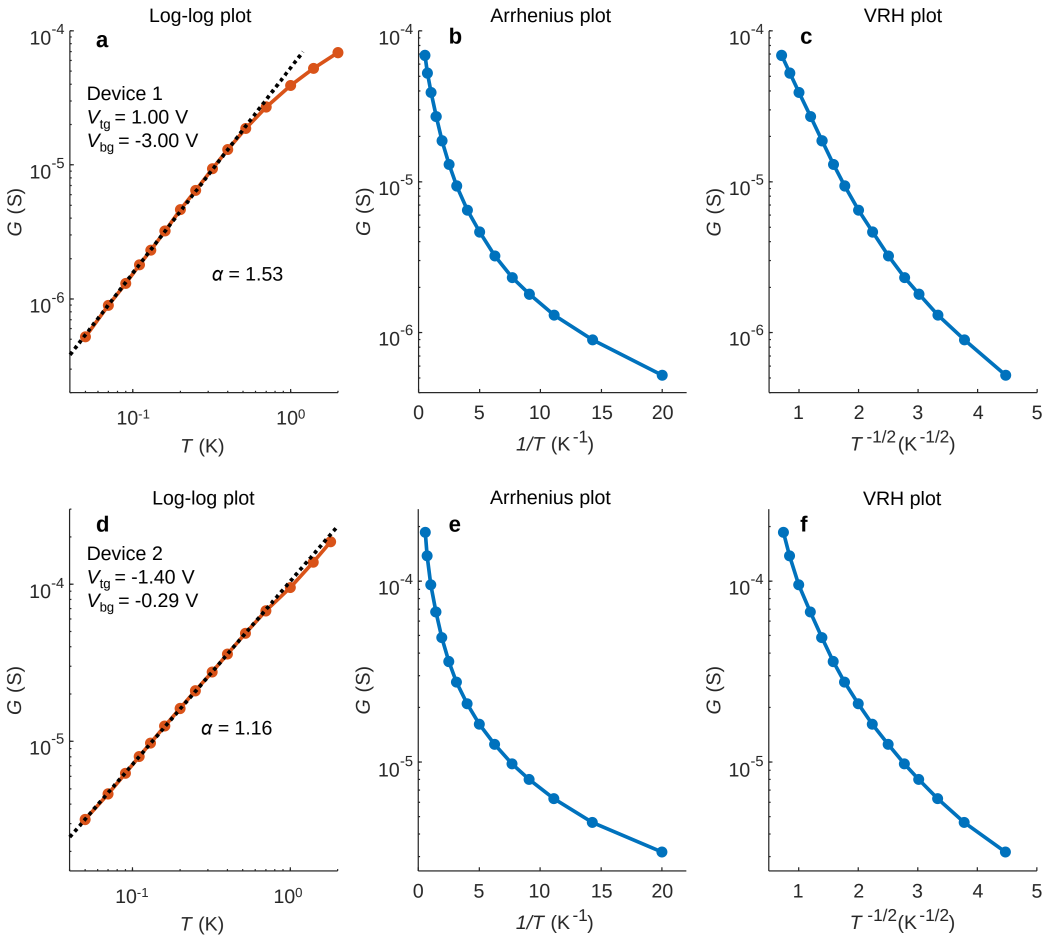

Supplementary Fig. 8 | Additional plots of conductance data. **a**-**c**, the same T-dependent conductance data shown in Fig. 3a but displayed in a log-log plot (**a**), an Arrhenius plot (**b**), and a plot against inverse square root of T (**c**). For Luttinger Liquid physics (power-law behavior), it corresponds to $G \propto T^{\alpha}$; for a band insulator $G \propto \exp(-\Delta/2k_BT)$ and for variable range hopping (VRH) process[24] $G \propto \exp(-T^{-1/2})$. Among all three, we find that the power law provides a much better description for data in the low-T regime. **d**-**f**, the same plots as in **a**-**c**, but for data shown in Fig. 3d.

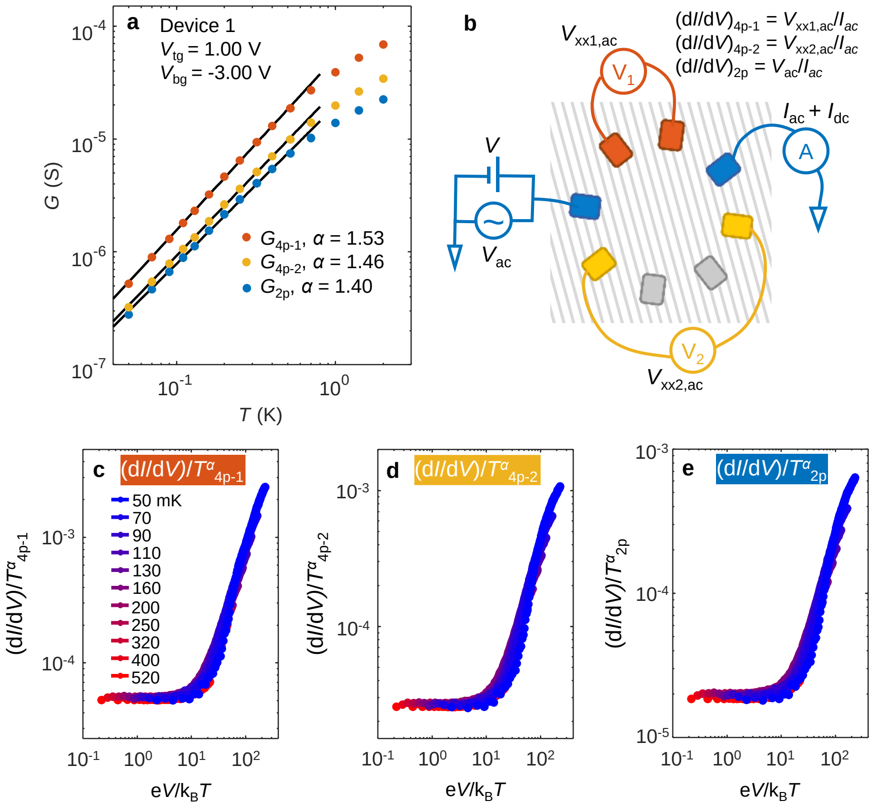

Supplementary Fig. 9 | Scaled conductance under different contact geometries. a, Conductance G *v.s.* T taken under the same gate configuration as that in Fig. 3 a-c (device 1), but plotting data with three different measurement geometries shown in **b**. The solids lines are the power law fits to the low-T data, all three data sets yield a very similar exponent. **b**, Measurement geometries for data in **a** and **c**-**e**. Orange data points in **a** correspond to a four-probe geometry (4p-1), with the source and drain contacts labeled in blue while voltage probes labeled in orange. Yellow data points in **a** (4p-2) correspond to the same source-drain contacts but with a different pair of voltage probes (labeled in yellow). Blue data points (2p) correspond to the same source and drain contacts and use the same source-drain contacts as the voltage probes (a two-probe geometry). **c-e**, Scaled conductance plots for the three corresponding measurement geometries. All follow excellent scaling behaviors and an exponent very close to each other.

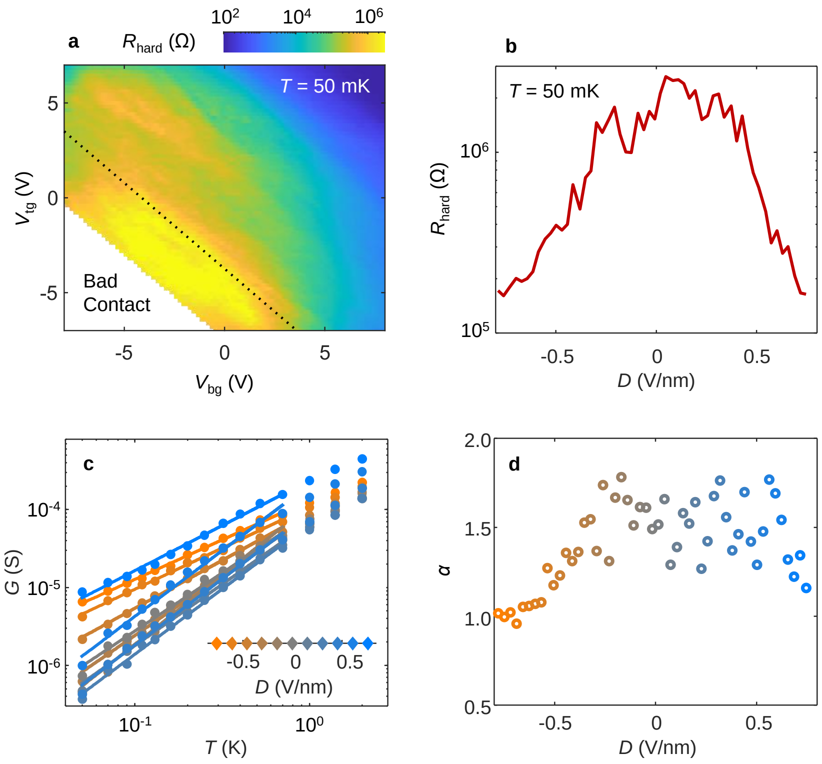

Supplementary Fig. 10 | Displacement field (*D*) effect on the LL behavior (Device 2). a, Four-probe resistance measured along the hard direction (R_{hard}) at 50 mK. The white color region is where bad contact prevents a reliable measurement at this temperature. **b**, R_{hard} *versus* D. Data are extracted from **a**, along the dotted line. **c**, Hard-direction conductance G ($\equiv 1/R_{hard}$) *versus* T, at different D along the dotted line shown in **a**. D for each colored curve is indicated in the insert. **d**, D-dependent power law component α, extracted from **c**. It appears that the LL behavior is very sensitive to n_g, but not D. At high D ($|D| > 0.5$ V/nm) a drop in α is seen, potentially indicating a transition especially if D is further increased.
